# Editorial: Receptor biology and cell signaling in diabetes: volume II

**DOI:** 10.3389/fphar.2023.1274914

**Published:** 2023-09-13

**Authors:** Swayam Prakash Srivastava, Keizo Kanasaki

**Affiliations:** ^1^ Hartman Institute of Therapeutic Organ Regeneration, Division of Regenerative Medicine, Department of Medicine, Weill Cornell Medicine, New York, NY, United States; ^2^ Internal Medicine 1, Shimane University Faculty of Medicine, Izumo, Japan

**Keywords:** endothelial cells, diabetes, diabetic kideny disease, endothelial dysfuction, receptor biology, sirtuin3 (sirt3), glucocorticoid receptor, fibroblast growth factor receptor 1 (FGFR1)

## Introduction

Approximately one-third of diabetic patients eventually develop kidney dysfunction, a condition that is associated with kidney fibrosis (Thomas et al., 2015; Srivastava and Kanasaki, 2022). Kidney fibrosis is characterized by excess deposition of extracellular matrix, accumulation of fibrillar collagen, activated myofibroblasts, and inflammatory cells in the diabetic kidney. Activation of transforming growth factor-β receptors (TGFβRs) and Wnt receptors have been shown to induce mesenchymal activations in many cell types (Yuan et al., 2022). Several factors, such as interactions between dipeptidyl peptidase-4 (DPP-4) and integrin β1, induce TGFβRs heterodimerization and related fibrogenesis in diabetic endothelial cells (DECs) (Shi et al., 2015). In contrast, endothelial glucocorticoid receptors (GRs) mitigate mesenchymal activation not only in endothelial cells but also in tubules by inhibiting aberrant Wnt signaling, and fibroblast growth factor receptor 1 (FGFR1) is a critical anti-EndMT molecule in DECs (Li et al., 2017; Srivastava et al., 2021d; Srivastava and Goodwin, 2023). A better understanding of receptor biology and cell signaling in diabetes and related complications is urgently needed to catalyze the development of new therapeutics. Several pharmacological interventions, such as dipeptidase transferase-4 (DPP-4) inhibitors, mineralocorticoid receptor antagonists, N-acetyl-seryl-aspartyl-lysyl-proline, and endothelin receptor antagonists, are beneficial in the treatment of diabetes and related kidney diseases (Kanasaki et al., 2014; Nagai et al., 2014; Srivastava et al., 2021a). In this context, sodium-glucose cotransporter −2 (SGLT-2) inhibitors have emerged as potential drugs to combat diabetic kidney disease (DKD). We have identified new chemical entities and pharmacophores that improve receptor-mediated altered cell signaling in different cell types. This manuscript describes the essential role of GR, sirtuin 3 (SIRT3), and FGFR1 signaling in DECs, which are known to inhibit defective central metabolism-linked endothelial-to-mesenchymal transitions (EndMT) in diabetic kidneys and hearts, whereas DPP-4 and low-density lipoprotein receptor-related proteins 5 and 6 are key mesenchymal inducers in diabetic tubules and DECs.

In this Research Topic of Frontiers in Pharmacology, we discuss receptor dysfunction in different cell types and how targeting receptor dysfunction is driving therapies against kidney fibrosis and vascular dysfunction in diabetes.

We focused on two main sections.

## Receptor biology and regulation in the diabetic endothelium

Endothelial dysfunction, such as endothelial cell leakage, participation in thrombus formation, and mesenchymal activation, are critical pathogenic phenotypes in diabetes. EndMT is one of the mechanisms by which endothelial cells lose their endothelial cell features and acquire the characteristics of mesenchymal phenotypes (Srivastava et al., 2019). However, the regulatory control of endothelial cell homeostasis in diabetic nephropathy and cardiovascular disease is less investigated. Recent research has predominantly focused on the identification of key endogenous molecules that are linked to endothelial cell homeostasis. Using state-of-the-art technology, we have identified three essential molecules that are important for endothelial cell health: 1) endothelial glucocorticoid receptors (GRs), nuclear receptors, and their deficiency cause triggering of Wnt-associated mesenchymal activation in endothelial cells themselves and also in neighboring cells, resulting in severe fibrogenic responses in diabetic kidneys and hearts (Srivastava et al., 2021c; Srivastava et al., 2021d; Srivastava and Goodwin, 2023). 2) Endothelial FGFR1, a cell surface receptor, and its deficiency cause activation of mesenchymal mechanisms by downregulating gene expression levels of antifibrotic microRNAs (Srivastava et al., 2016; Li et al., 2020). Importantly, N-seryl-acetyl-lysyl-proline, a discovered antifibrotic peptide, performs its action by binding to FGFR1 (Srivastava et al., 2020b). 3) Endothelial SIRT3, a mitochondrial protein, regulates endothelial cell health by regulating metabolic flux through the modulation of pyruvate kinase M2 tetramer-to-dimer formation (Srivastava et al., 2020c; Srivastava et al., 2021b). Non-proliferating kidney tubules and endothelial cells are primarily dependent on fatty acid oxidation for their energy expenditure (Kang et al., 2015; Lovisa and Kalluri, 2018). However, loss of endothelial SIRT3 disrupts central metabolism and accelerates metabolic shifts in myofibroblasts, and these accumulative effects lead to mesenchymal activation and fibrosis in diabetic kidneys and hearts (Srivastava et al., 2018; Srivastava et al., 2021b; Liu et al., 2021). In addition, a few proteins have been identified, such as DPP-4, LRP5, and LRP6—these are mesenchymal inducers (Shi et al., 2015; Srivastava et al., 2020a). Therefore, endothelial GR, SIRT3, and FGFR1 are the essential molecules for endothelial cell health, while DPP-4, LRP5, and LRP6 are the mesenchymal molecules in diabetes. Targeting these molecules could be a potential therapeutic approach to combating endothelial dysfunction in diabetes.

In this Research Topic, Cao et al. describe cellular phenotypic switching and its association with renal fibrosis. The authors discussed the different types of kidney cells, for example, endothelial cells that undergo activation and differentiation processes and are reprogrammed to express markers of mesenchymal cells or podocyte-like cells, highlighting the molecular pathways involved in the cell-to-cell transition processes, which would provide valuable information for the design of effective therapies for DKD.

## Receptor biology and cell signaling in diabetes and related complications

The Epidemiology of Diabetes Interventions and Complications study highlights that poor initial glycemic control is associated with a high prevalence of future diabetic complications (Yahaya et al., 2023). This phenomenon has been termed “metabolic memory.” Hyperglycemia-derived metabolites that accumulate abnormally in organs cause diabetic complications.

In this Research Topic, Taguchi and Fukami report on the receptor for advanced glycation end products (RAGE), which is a multiligand receptor that is sequentially expressed in the body. The expression level of RAGE is upregulated in diabetic patients with hypertension, obesity, and chronic inflammation, suggesting that RAGE activation is a common cause for the development of DKD. Another study in this Research Topic by Pan et al. reviews the significance of adipose tissue thermogenins and targetable receptors. In their work, the authors discuss the functions of antidiabetic medications with known thermogenic mechanisms and focus on various receptor signaling pathways that can potentially be targeted to combat obesity and related diabetes.

Another study describes the critical role of GRP78 in regulating TGFβ1-mediated profibrotic responses via TSP1 in DKD. The data suggest an important function for csGRP78 in regulating high glucose-induced TSP1 transcriptional induction via PI3K/Akt signaling, and inhibition of csGRP78 signaling represents a novel therapeutic approach against renal fibrosis in DKD Trink et al. A review by Nakamura et al. describes the association between mTOR signaling and renal metabolism. This review elucidates that mTOR plays an important role in regulating renal gluconeogenesis, and its impairment may further contribute to hyperglycemia in T2D. Moreover, further research is required in this area to explain this theory.

## Conclusion

In this Research Topic, we have discussed the crucial receptors and related key cellular pathways to highlight future possible therapeutics against fibrogenesis in diabetes. The information gained from this Research Topic will be useful for clinicians and scientists in the development of novel therapeutics, new research approaches, and future research directions.
